# Supplementary material for: A Universal LC-MS/MS Method for Simultaneous Detection of Antibiotic Residues in Animal and Environmental Samples
Source: Antibiotics (Basel). 2022 Jun 24;11(7):845. doi: 10.3390/antibiotics11070845 (PMC9311993; doi:10.3390/antibiotics11070845)
Supplement: Supplementary file 1 [file antibiotics-11-00845-s001.zip › antibiotics-1762284-supplementary.pdf]

Table S1. Reduction in sensitivity of overall protocol for 43 antibiotics at spike-in concentrations of 10 μg, 5 μg, and 1 μg.

		Water Sample			Fecal Sample			Meat Sample		
**Groups**	**Antibiotics**	**10 μg spiked in**	**5 μg spiked in**	**1 μg spiked in**	**10 μg spiked in**	**5 μg spiked in**	**1 μg spiked in**	**10 μg spiked in**	**5 μg spiked in**	**1 μg spiked in**
Aminoglycosides	Gentamicin	0	0	0	0	0	0	0	0	0
	Kanamycin sulfate	0	0	0	0	0	0	0	0	0
	Neomycin trisulfate salt hydrate	0	0	0	0	0	0	0	0	0
	Spectinomycin hydrochloride pentahydrate	100	66.7	0	66.7	66.7	0	100	100	0
	Streptomycin sulfate salt	66.7	33.3	0	100	0	0	100	66.7	0
Amphenicols	Chloramphenicol	100	100	0	100	100	0	100	100	66.7
	Florfenicol	100	100	100	100	100	100	100	100	100
Antifolate	Trimethoprim	100	100	100	100	100	100	100	100	100
Carbapenems	Meropenem	0	0	0	0	0	0	0	0	0
Cephalosporins	Cefalexin	100	100	100	100	100	100	100	100	100
	Cefquinome sulfate	0	0	0	0	0	0	0	0	0
	Ceftazidime	0	0	0	0	0	0	0	0	0
	Ceftiofur sodium	100	100	66.7	100	100	66.7	100	100	0
	Cefuroxime	0	0	0	0	0	0	0	0	0
Fluoroquinolones	Ciprofloxacin	0	0	0	0	0	0	0	0	0
	Enrofloxacin	100	100	100	100	100	100	100	100	100
	Levofloxacin	100	100	100	100	100	100	100	100	100
	Norfloxacin	0	0	0	0	0	0	0	0	0
	Ofloxacin	100	100	100	100	100	100	100	100	100
Glycopeptides	Vancomycin	0	0	0	0	0	0	0	0	0
Lincosamides	Clindamycin phosphate	100	100	100	100	100	100	100	100	100
	Lincomycin hydrochloride	100	100	100	100	100	100	100	100	100
Macrolides	Erythromycin	100	100	33.3	100	33.3	0	100	100	33.3
	Tilmicosin	66.7	66.7	0	100	100	33.3	100	100	33.3
	Tylosin tartrate salt	100	100	100	100	100	100	100	100	66.7
	Tylvalosin	100	100	33.3	100	100	100	100	100	100
Nitroimidazole	Metronidazole	100	100	100	100	100	33.3	100	100	66.7

Penicillins	Amoxicillin	0	0	0	0	0	0	0	0	0
	Ampicillin	100	100	66.7	100	100	0	100	100	33.3
	Penicillin G sodium salt	100	100	0	100	33.3	0	100	100	0
Pleuromutilins	Tiamulin	100	100	100	100	100	100	100	100	100
Polymyxins	Colistin	0	0	0	0	0	0	0	0	0
Quinoxaline 1,4-di-N-oxides (QdNOs)	Mequindox	100	100	100	100	100	100	100	100	100
Sulfonamides	Sulfachloropyridazine	100	100	100	100	100	100	100	100	100
	Sulfadiazine	100	100	100	100	100	100	100	100	100
	Sulfadimidine	100	100	100	100	100	100	100	100	100
	Sulfamethoxazole	100	100	100	100	100	100	100	100	100
	Sulfamonomethoxine	100	100	100	100	100	100	66.7	100	100
Tetracyclines	Chlortetracycline hydrochloride	100	100	100	100	100	100	100	100	66.7
	Doxycycline	100	100	0	100	100	33.3	100	100	0
	Oxytetracycline	100	100	100	100	100	100	100	100	100
	Tetracycline	100	100	100	100	100	100	100	100	66.7

Sensitivity = [number of true positives/(number of true positives + number of false negatives)] × 100%. Pink: an indication of a reduction in sensitivity for antibiotic as compared to previous spike-in concentration for the same sample type.
